# Type I and Type III Interferons Drive Redundant Amplification Loops to Induce a Transcriptional Signature in Influenza-Infected Airway Epithelia

**DOI:** 10.1371/journal.ppat.1003773

**Published:** 2013-11-21

**Authors:** Stefania Crotta, Sophia Davidson, Tanel Mahlakoiv, Christophe J. Desmet, Matthew R. Buckwalter, Matthew L. Albert, Peter Staeheli, Andreas Wack

**Affiliations:** 1 Division of Immunoregulation, MRC National Institute for Medical Research, The Ridgeway, London, United Kingdom; 2 Department of Virology, University of Freiburg, Freiburg, Germany; 3 Laboratory of Cellular and Molecular Immunology, Interdisciplinary Cluster of Applied Genoproteomics (GIGA) Research Center and Faculty of Veterinary Medicine, University of Liege, Liege, Belgium; 4 Unité Immunobiologie des Cellules Dendritiques, Department of Immunology, Institut Pasteur, Paris, France; 5 INSERM U818, Paris, France; 6 Université Paris Descartes, Paris, France; 7 Spemann Graduate School of Biology and Medicine (SGBM), Albert Ludwigs University Freiburg, Freiburg, Germany; University of Wisconsin-Madison, United States of America

## Abstract

Interferons (IFNs) are a group of cytokines with a well-established antiviral function. They can be induced by viral infection, are secreted and bind to specific receptors on the same or neighbouring cells to activate the expression of hundreds of IFN stimulated genes (ISGs) with antiviral function. Type I IFN has been known for more than half a century. However, more recently, type III IFN (IFNλ, IL-28/29) was shown to play a similar role and to be particularly important at epithelial surfaces. Here we show that airway epithelia, the primary target of influenza A virus, produce both IFN I and III upon infection, and that induction of both depends on the RIG-I/MAVS pathway. While IRF3 is generally regarded as the transcription factor required for initiation of IFN transcription and the so-called “priming loop”, we find that IRF3 deficiency has little impact on IFN expression. In contrast, lack of IRF7 reduced IFN production significantly, and only IRF3^−/−^IRF7^−/−^ double deficiency completely abolished it. The transcriptional response to influenza infection was largely dependent on IFNs, as it was reduced to a few upregulated genes in epithelia lacking receptors for both type I and III IFN (IFNAR1^−/−^IL-28Rα^−/−^). Wild-type epithelia and epithelia deficient in either the type I IFN receptor or the type III IFN receptor exhibit similar transcriptional profiles in response to virus, indicating that none of the induced genes depends selectively on only one IFN system. In chimeric mice, the lack of both IFN I and III signalling in the stromal compartment alone significantly increased the susceptibility to influenza infection. In conclusion, virus infection of airway epithelia induces, via a RIG-I/MAVS/IRF7 dependent pathway, both type I and III IFNs which drive two completely overlapping and redundant amplification loops to upregulate ISGs and protect from influenza infection.

## Author Summary

The response of cells to virus infection depends on Interferons (IFNs), a group of cytokines which activate the expression of hundreds of genes that help control viral replication inside infected cells. While type I IFN was discovered in 1957, type III IFN (IFNλ, IL-28/29) was characterized recently and is known for its role in the response to hepatitis C virus. Airway epithelia are the primary target of influenza virus, and we studied how infection induces IFNs and which IFN is most important for the epithelial anti-influenza response. We found that infected epithelia detect virus through the cytoplasmic RIG-I/MAVS recognition system, leading to activation of the transcription factor IRF7 and subsequent induction of both type I and III IFNs. All ensuing cellular responses to infection are dependent on the production and secretion of IFNs, as responses are lost in epithelia lacking receptors for both type I and III IFNs. Finally, gene induction is indistinguishable in single receptor-deficient and wild-type cells, indicating that the two IFN systems are completely redundant in epithelia. Thus, influenza infection of airway epithelia induces, via a RIG-I/MAVS/IRF7 dependent pathway, both type I and III IFNs which drive two overlapping and redundant amplification loops to upregulate antiviral genes.

## Introduction

The type I interferon family is a group of cytokines encoded by a single IFNβ gene and a tandem cluster of multiple IFNα genes that were first characterized for their ability to interfere with influenza virus replication [1] and are now recognized as powerful inducers of the host response to viral infections.

IFN induction by influenza A virus (IAV) depends on recognition of viral components by either cytoplasmic receptors or the Toll-like receptor (TLR) system, depending on the infected cell type. While plasmacytoid dendritic cells (pDC) use TLR7 to recognize influenza virus, in fibroblasts and conventional DCs IFNβ induction requires recognition of RNA viral genomes by the cytoplasmic RNA helicase retinoic acid-induced gene I (RIG-I) [2], [3]. Upon RNA binding, RIG-I interacts with the mitochondrial adaptor protein MAVS (also known as IPS-1, CARDIF and VISA) and initiates a signalling cascade that culminates in the activation of the transcriptional factors AP-1, NF-κB and IRF3, and the expression of IFNβ and IFNα4 in mouse (or IFNα1 in humans) [4]–[7].

Once secreted, IFNβ and IFNα4 acts in both a paracrine and autocrine way through binding to the ubiquitously expressed heterodimeric IFNα/β receptor (IFNAR1/2) to induce activation of the receptor–associated tyrosine kinases JAK1 and Tyk2 and subsequent phosphorylation of the transcriptional factors STAT1 and STAT2 [8]. Activated STATs then form transcription factor complexes, including STAT1 homodimers and a STAT1/STAT2/IRF9 heterotrimer known as ISGF3 [9], and mediate the induction of hundreds of IFN-stimulated genes (ISGs), whose expression determines the establishment of an antiviral state inside the cell.

Recently, a novel group of IFNs was described and named type III IFNs. This new IFN family has three members: IFNλ1 (a pseudogene in mouse), IFNλ2 and IFNλ3, alternatively named IL-29, IL-28A and IL-28B respectively [10], [11].

IFNλ induction depends on the same triggers and signalling pathways that regulate type I IFN expression [12], [13], with the RIG-I/MAVS/TBK1/IRF3 axis being particularly relevant in mouse embryonic fibroblasts (MEFs) upon viral infection [14].

IL-28/29 act through a distinct receptor complex consisting of IL-28Rα, specific for type III IFNs, and the IL-10Rβ chain, which is also part of the receptors for IL-10, IL-22 and IL-26. Despite activating different receptors, both type I and III IFNs activate the JAK-STAT signalling pathway, that leads to the formation of the ISGF3 complex [15] and the induction of ISGs.

The most important determinant of the different biological activities of IFNα/β and IFNλ is the distribution of their receptors. While the receptor for type I IFNs is expressed on all cells, IL-28Rα is found primarily on epithelial cells of both the respiratory and the gastrointestinal tract [16]–[18]. This finding suggests that type III IFNs may act in a cell-type restricted manner and may selectively contribute to the innate immunity of mucosal surfaces, potential entry sites for many pathogenic viruses.

The airway epithelium is a pseudostratified, columnar epithelium consisting of ciliated, basal and secretory goblet cells, that lies at the interface between the host and the environment and provides the first line of defence against inhaled microorganisms. Airway epithelial cells represent the target of many respiratory viruses, including Influenza virus, Adenovirus, Rhinovirus and RSV [19]. As epithelial cells express both cell surface and endosomal pattern recognition receptors (PRRs) and intracellular viral sensors, they can promptly detect invading microbes and react by producing cytokines, chemokines and antimicrobial peptides, thus initiating inflammatory and immune responses [20], [21].

While the PRRs and downstream signals required for influenza ISG induction have been mapped out in detail in other cell types, it is less clear which recognition systems are in action in airway epithelia. Moreover, while the importance of type III IFNs in epithelial responses has been documented, it is unclear whether the signatures induced by interferon type I or III overlap and which, if any, ISG subsets are selectively induced by one or the other.

To address these issues, we established cultures of primary differentiated murine tracheal epithelial cells (MTEC) and, using a genetic approach together with microarray analysis, investigated the mechanisms that lead to IFN induction in response to influenza A infection. We also assessed the relative contribution of type I and III IFNs to the establishment of an antiviral state by comparing the pattern of influenza-induced gene expression in the absence of either IFNα/β signalling, IFNλ signalling or both. These studies help define the biology and nature of the antiviral state induced by different IFNs in primary cells and determine whether and which genes are still induced by influenza infection in the complete absence of both IFN type I and III signalling.

## Results

### In MTEC, the induction of interferons requires MAVS and virus replication

**Figure 1:**
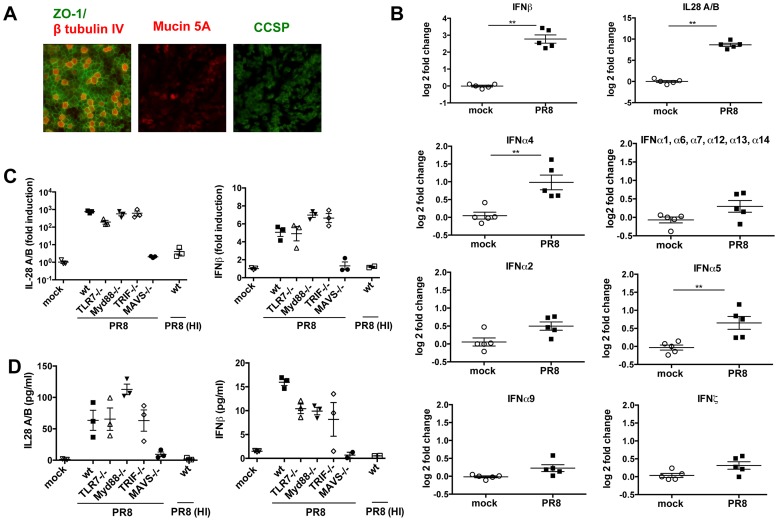
In primary MTEC, type I and III IFNs are induced upon influenza virus A infection, in a MAVS and replication dependent way. (A) Mouse tracheal epithelial cells were costained for ZO-1 (green) and β tubulin IV (red), or Mucin 5A (red) and Clara cell secretory protein (CCSP) (green). Images were acquired at ×20 magnification. (B) Total RNA from mock infected and PR8 infected (moi = 0.3) MTEC was analysed using Affymetrix Mouse Genome 430 2.0 microarrays. The signal intensity of each probe was first normalized on the median intensity of that probe across the control group and then represented as log2 fold change relative to the controls. Asterisks indicated statistically significant differences (unpaired t test; **, p<0.01). (C) qRT-PCR analysis of IL-28A/B and IFNβ1 transcripts of MTEC derived from wild-type, TLR7^−/−^, MyD88^−/−^, TRIF^−/−^ and MAVS^−/−^ mice, mock infected or infected with either PR8 or heat inactivated PR8. Fold induction is relative to mock treated samples at 24 hpi +/− SEM. (D) IL-28A/B level in the supernatants of the indicated cultures were measured by ELISA 24 hpi.

Primary MTEC were grown to confluence and exposed to air for 14 days, leading to formation of a fully differentiated, polarized epithelium containing ciliated and secretory goblet and Clara cells (Fig. 1A), a cellular composition which closely matches that of airway epithelia in vivo. These differentiated cultures were then infected with IAV, at a multiplicity of infection (moi) of 0.3. Intracellular FACS analysis of the infected cultures showed that approximately 10% of MTEC were expressing viral nucleoprotein (NP) 24 hours post infection (hpi) (Fig. S1). Total RNA was isolated from five replicate cultures 24 hpi and analyzed using microarrays. We first investigated which type of IFN was induced in response to the infection. To this purpose, after normalizing the signal intensity of each probe in each sample to the median intensity of that probe in the control group, and filtering for genes that were expressed above background level, we searched for the probe sets which represent IFNs in the data set. As shown in Fig. 1B, both type I (IFNβ, α4 and α5) and type III IFNs (IL-28A/B) were significantly induced in the infected samples, with IL-28A/B being the most strongly upregulated. The induction of all other IFNα genes did not reach statistical significance. Type II IFN (IFNγ) did not pass the initial filtering for genes expressed above background and was not induced upon infection (not shown). To identify the pattern recognition receptors responsible for IFN induction in this experimental model, we infected MTEC cultures from wild-type and knock-out mice and analyzed the expression of both IFNβ and IL-28 by quantitative RT-PCR and ELISA. While in some immune cells TLR7 is the major PRR mediating IFN induction by influenza virus [22], we found that the absence of TLR7 or its downstream adaptor MyD88 had no impact on the induction of IFNs in influenza-infected epithelia. Similarly, the adaptor TRIF used by TLR3 and TLR4 is not required for IFN induction in epithelia. In contrast, MTEC deficient in MAVS were unable to produce IFNs in response to influenza infection, suggesting involvement of the RIG-I pathway (Fig. 1C, 1D). We next sought to determine whether virus replication is required for the induction of IFNs. Treatment of PR8 at 65°C inactivates the viral polymerase and prevents viral replication, without abrogating virus attachment to the cells (Fig. S2A, S2B). This treatment abolished the virus' ability to induce IFNs (Fig. 1C, 1D) indicating that IAV virus access to the cytoplasm and subsequent replication are required to initiate an IFN response in MTEC.

### An IFN transcriptional signature is induced upon infection

**Figure 2:**
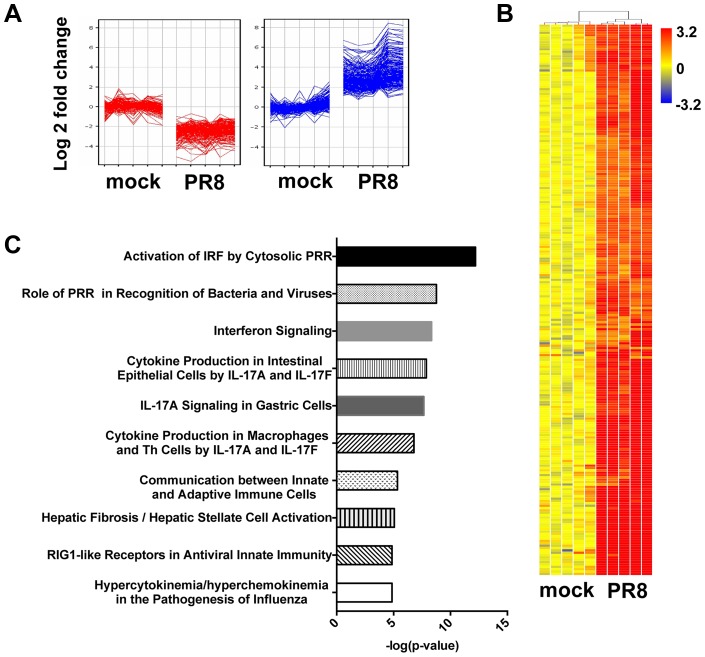
In primary MTEC, influenza A infection induces an IFN signature. Total RNA from mock and PR8 infected cells was analysed using Affymetrix Mouse Genome 430 2.0 microarrays at 24(≥4-fold change relative to mock infected; t-test unpaired, p<0.01, Benjamini-Hochberg multiple test correction). (A) K-means clustering of the differentially expressed genes. (B) Heat map of the 234 upregulated-gene, as shown in (A). The range of fold changes is expressed in a log2 scale. (C) Ingenuity Pathway Analysis of the upregulated genes in influenza infected MTEC.

To identify the transcripts that were differentially expressed in infected MTEC cultures at 24 hpi, we performed a supervised analysis under stringent conditions (≥4-fold change relatively to mock infected samples, t-test unpaired p value <0.01, Benjamini-Hochberg multiple statistical correction). The differentially expressed genes were then partitioned by K-means clustering (Fig. 2A) into 2 groups (177 downregulated genes; 234 upregulated genes), and the 234 upregulated genes were hierarchically clustered to generate the heat map in Fig. 2B. Ingenuity Pathway Analysis of the list of upregulated genes confirmed “activation of interferon regulated factors by cytosolic PRR” and “interferon signalling” as two overrepresented pathways in the infected samples (Fig. 2C). Moreover, 80 (35%) of the upregulated genes were recognized as interferon stimulated gene by the INTERFEROME database.

### IRF3 is dispensable for IFN induction

**Figure 3:**
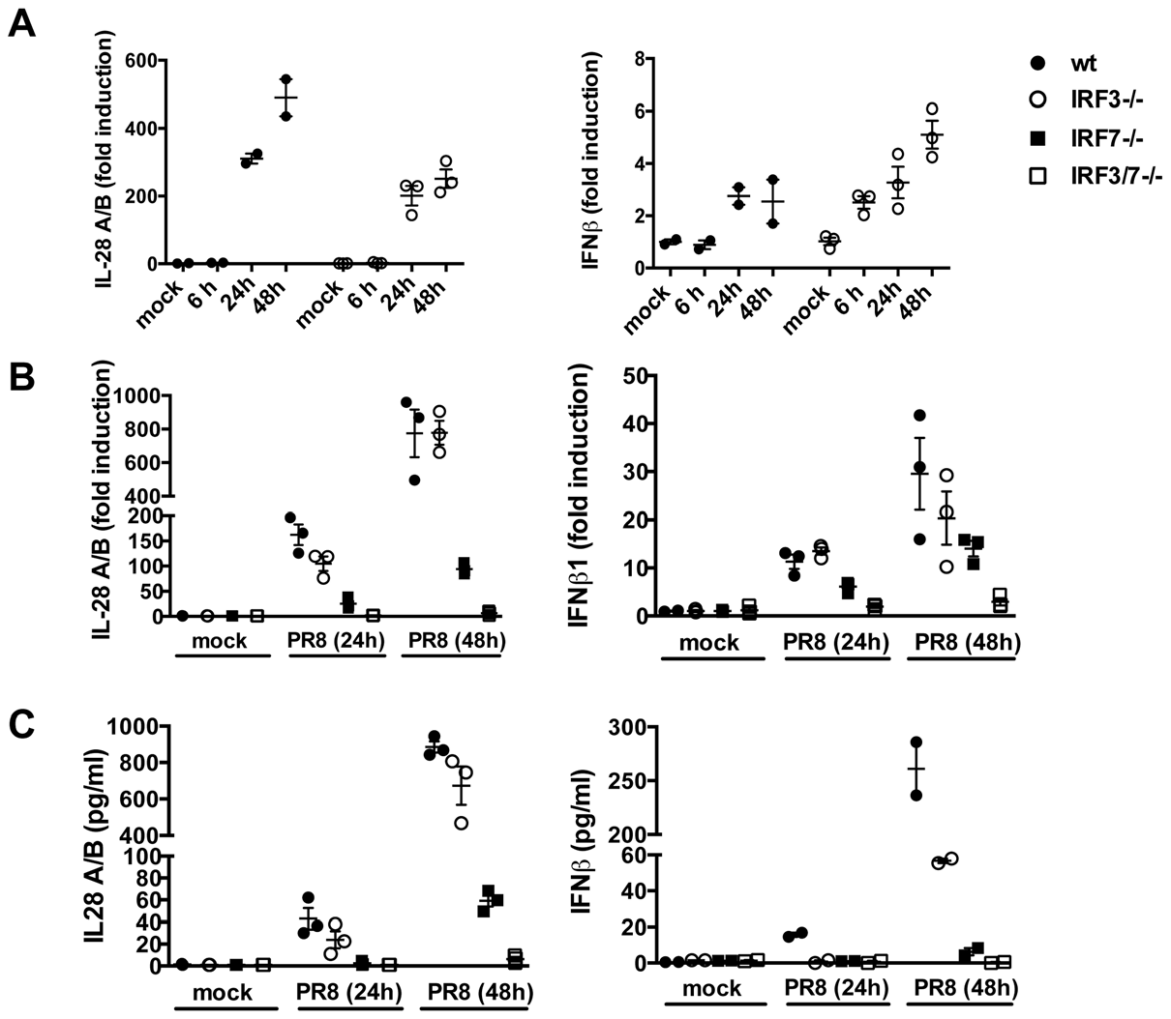
IRF3 is not required for IFN induction in primary MTEC. (A) MTEC derived from wild-type or IRF3^−/−^ mice were mock treated or infected with PR8. RNA was analyzed at the indicated time by qRT-PCR, normalized to the amount of HPRT transcripts and measured as fold induction relative to the level in the corresponding control samples. (B) Expression of IL-28A/B and IFNβ was measured by qRT-PCR at 24 hpi, in epithelial cultures of the indicated genotypes. (C) Protein levels of IL-28A/B in the supernatants of mock and PR8 infected cells were measured by ELISA 24 hpi or, where indicated, at 48 hpi. Error bars indicate the SEM of replicates.

A major level of control of IFN production depends on transcriptional regulation. The general paradigm for IFNβ induction involving recruitment of the transcription factors IRF3 and p50/RelA NF-κB has recently been shown to apply also to type III IFN induction, at least in MEFs [14]. Unexpectedly, IRF3 deficient MTEC were not impaired in their ability to upregulate both IL-28 and IFNβ in response to infection (Fig. 3A). In contrast, IRF7^−/−^ epithelia showed a marked reduction in the amount of IFN induced; however, the induction of both IL-28 and IFNβ was completely abolished only in doubly deficient IRF3^−/−^IRF7^−/−^ MTECs, as assessed by qPCR for gene expression and by ELISA for protein secretion (Fig. 3B, C).

### ISGs are induced in the absence of IFNs

**Figure 4:**
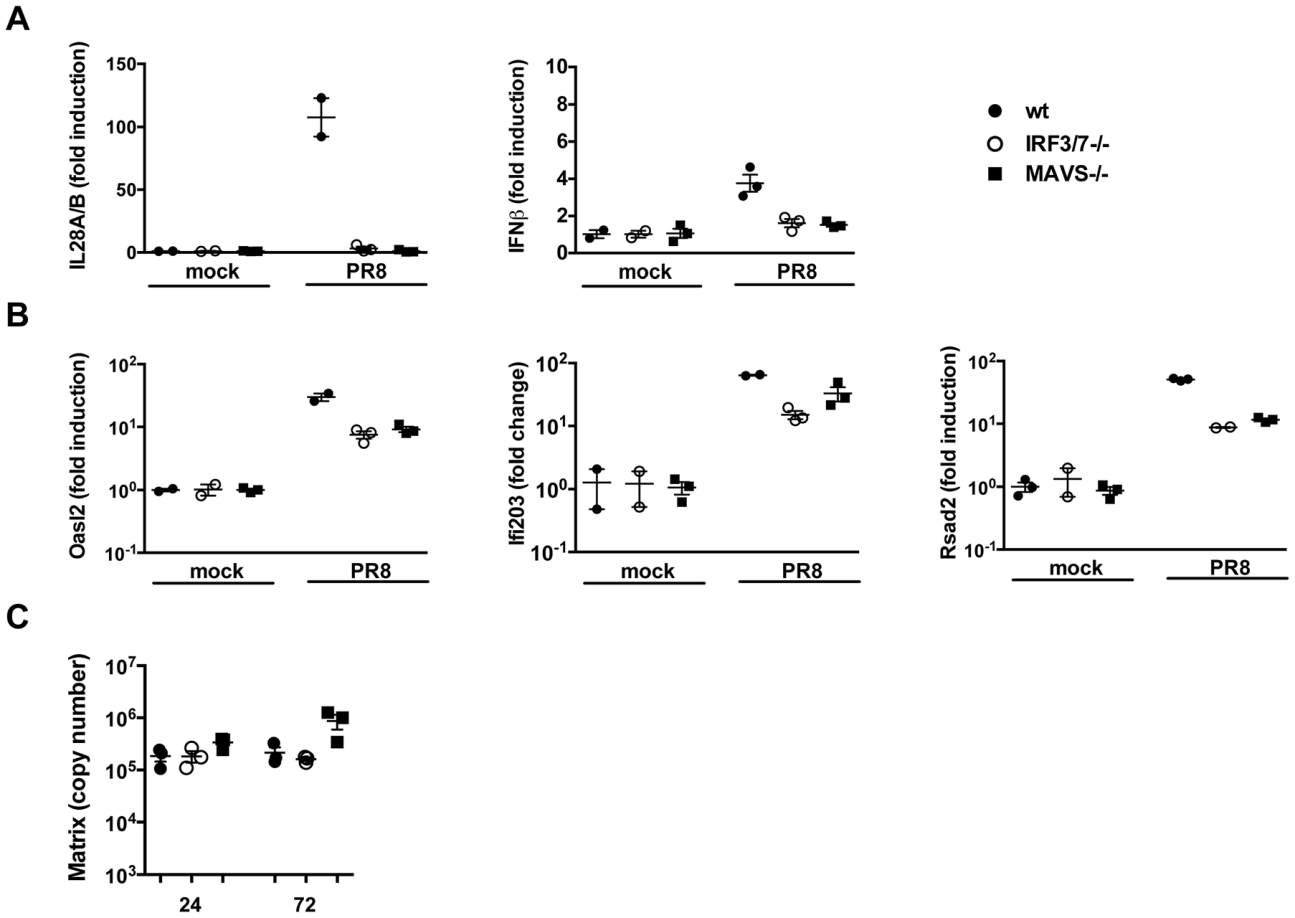
ISGs are induced in the absence of detectable type I and III IFNs. Wild-type, MAVS^−/−^ and IRF3^−/−^IRF7^−/−^ MTEC were either mock infected or infected with PR8 and their RNA analyzed 24 hpi for the induction of (A) both IFNs (IL-28A/B and IFNβ) and (B) ISGs (Oasl2, Ifi203, Rsad2). All the transcripts were first normalized to HPRT levels and then expressed as fold induction relative to the mean of mock infected controls, +/− SEM. (C) Viral replication was measured by qRT-PCR on the Influenza A Matrix gene and expressed as copy number +/− SEM.

Previous studies have demonstrated that the entry of enveloped viruses like HSV and VSV into fibroblast cells can lead to the induction of a subset of ISGs in an IFN-independent manner and that either IRF3 [23], [24], IRF7 [25] or IRF1 [26] may have functions that are redundant to that of ISGF3 and therefore induce an IFN-like transcriptome in the absence of IFN signalling. For these reasons, we sought to determine whether in airway epithelia, ISGs could be induced in the absence of IFNs. The data shown so far point to two situations, i.e. the MAVS^−/−^ and the IRF3^−/−^IRF7^−/−^ epithelia, in which no IFN production could be detected, both at the protein and at the RNA level (Fig. 1C, 1D, 3B, 4A). Surprisingly, in both conditions, infection with influenza virus led to the induction of most of the ISGs tested, albeit at lower levels than in wild-type epithelia (Fig. 4B and S3). This correlated with virus control as infected wild- type, MAVS and IRF3/7 deficient epithelia had similar virus titers over the course of infection (Figure 4C).

These results can be interpreted in two ways; first, following IAV infection, a subset of ISGs may be induced through a MAVS and/or IRF3/IRF7 independent pathway that does not require interferons. Alternatively, in MAVS^−/−^ and IRF3^−/−^IRF7^−/−^ cells, minute, steady state amounts of IFNs could still be produced and be sufficient to induce ISGs in a context of viral infection.

### ISG induction requires type I or III IFN signalling in the MTEC system

**Figure 5:**
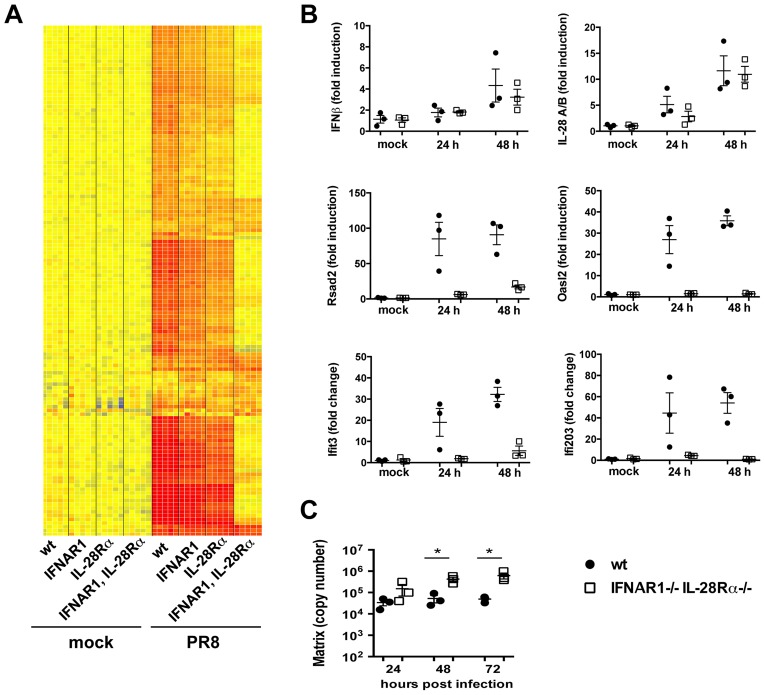
Type I and III are redundant in the MTEC system. (A) Total RNA from mock and PR8 infected cells was analysed using Affymetrix Mouse Genome 430 2.0 microarrays at 24 hpi. Supervised analysis was performed using statistical filtering (≥4-fold change relative to mock infected wild-type in at least one treatment group; 2-way ANOVA, p<0.01, Benjamini-Hochberg multiple test correction). (A) Heat map of the upregulated genes. (B) Quantitative RT-PCR analysis of RNA samples extracted from mock and PR8 infected wild-type and IFNAR1^−/−^IL-28Rα^−/−^ double knock-out MTEC at 24 and 48 hpi. All transcripts were first normalized to HPRT levels and then expressed as fold induction relative to the mean of mock infected controls, +/− SEM. (C) Wild-type and IFNAR1^−/−^IL-28Rα^−/−^ double knock-out MTEC were infected and RNA samples collected at the indicated time points. Viral replication was measured by qRT-PCR on the Influenza A Matrix gene and expressed as copy number +/− SEM. Asterisks indicate differences that are statistically significant (unpaired t test; *, P<0.05).

To test these alternative hypotheses, we infected epithelia deficient for either type I, type III or both IFN receptors and analysed their transcriptional response to influenza A virus by microarray analysis. RNA from five replicate samples were first normalized to the median of mock infected samples and then filtered on expression (20–100^th^ percentile in at least 50% of samples). A supervised analysis under stringent conditions (≥4-fold change versus wild-type mock in at least one infected group, 2-way ANOVA, p value of <0.01, Benjamini-Hochberg multiple statistical correction) and k-means clustering led to a list of 136 upregulated genes and a list of 50 downregulated genes (not shown). The induced genes were then hierarchically clustered to generate the heat map in Fig. 5A.

Influenza infection of IFNAR1^−/−^IL-28Rα^−/−^ double knock-out epithelia induced the expression of IFNβ and IL-28A/B at levels comparable to the wild-type controls even at later time points during an infection (Fig. 5B), indicating that these genes are most likely upregulated directly downstream of the RIG-I/MAVS pathway and do not require IFN-driven positive feed-back on themselves. In contrast, IFNAR1^−/−^IL-28Rα^−/−^ cells have lost the ability to upregulate many of the genes that were induced in the wild-type control (Fig. 5A), including known ISGs such as Rsad2, Oasl2 and others (Fig. 5B).

To analyse more globally the 136 up-regulated genes shown in Fig. 5A, they were further partitioned by K-means clustering into those that were not induced in infected IFNAR1^−/−^IL-28Rα^−/−^ cells (110 “IFN-dependent” genes, Fig. S4C) and those that were still induced (26 “IFN-independent” genes, Fig. S4A, B): analysis by the INTERFEROME database scored 58 (53%) of the “IFN-dependent” genes as ISGs. The “IFN-independent” genes comprise a smaller group of genes, including many chemokines (Fig. S4B). Although 5 (23%) of these genes (CXCL1, CXCL3, CSF-2, CXCL5 and CD274) were identified as ISGs by the INTERFEROME database, it has been described elsewhere that their expression can be also induced independently of IFNs, most likely through regulation by transcription factors like NF-kB, PPARγ and GATA-1 [27]. The mechanism by which they are induced by IAV in our system is currently under investigation.

The transcriptional signatures obtained for the single IFNAR1 and IL-28Rα knock-outs were very similar to the one for cells of wild-type origin (Fig. 5A). To address directly whether induction of some ISGs specifically depends on one type of IFN, we filtered the list of 136 infection-induced genes in Fig. 5A for genes that differ between either the wild-type and single knock-outs or between the two single knock-outs (Fig. S5). Indeed, only 11 genes were found, and in most cases, genes were induced in all three genotypes although at lower intensity in the single knock-outs.

Overall, our results indicate that in airway epithelia, the induction of an antiviral state depends on either type I or type III IFN signalling. Both types of IFN independently drive parallel, completely redundant amplification loops, each leading to the induction of the same set of genes. In the absence of both receptors, the IFN signature disappears almost entirely, indicating that no other mechanism can replace the IFN loop for the induction of ISGs.

Importantly, the lack of ISG induction in IFNAR1/IL28Rα deficient epithelia has biologic consequences as it leads to significantly higher virus titers at later points during infection (Figure 5C). No significant differences in viral titers were seen between wild-type and either IFNAR1 or IL-28Rα single knock-out.

**Figure 6:**
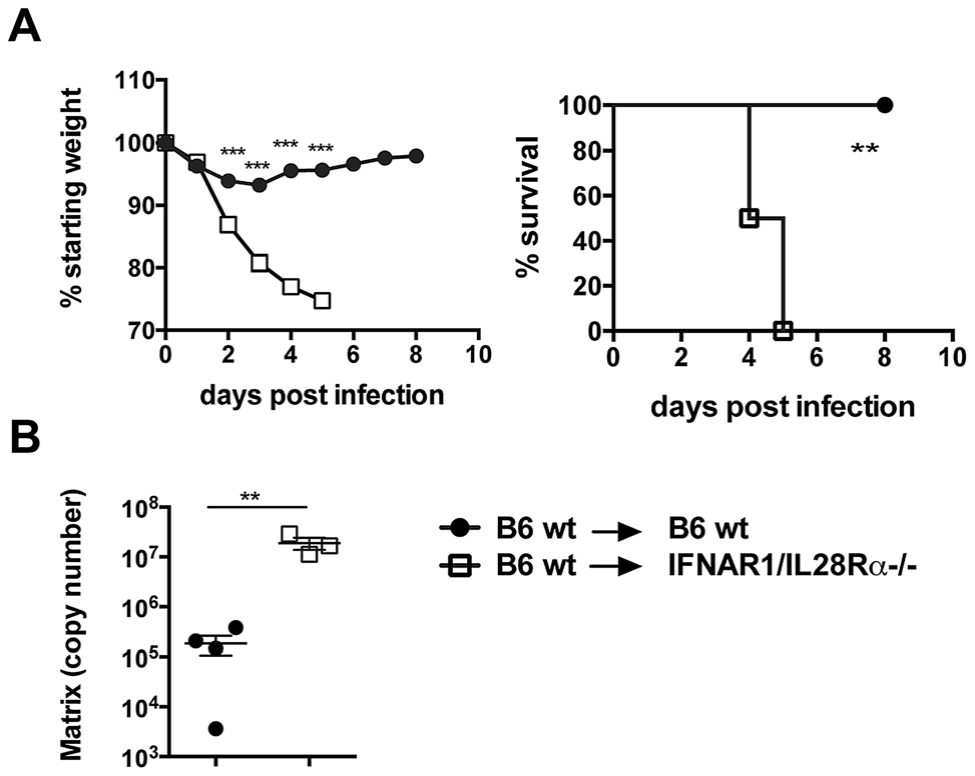
Lack of type I and III IFN signalling in the stromal compartment increases susceptibility to IAV infection. Chimeric mice were infected i.n. with 10^5^ TCID_50_ PR8, (A) Weight loss and mortality were measured. Graphs show mean ± SEM and are representative of 2 independent experiments (n = 6). (B) Viral replication was assessed by qRT-PCR on total lung RNA at 4 days post infection. *** p<0.001, ** p<0.01 by 2-way ANOVA with Bonferroni post tests (weight loss), Log-rank (Mantel-Cox) Test (survival) or unpaired t test (RT-PCR quantification).

To test in vivo whether lack of IFN responsiveness in lung epithelia impacts the disease course of influenza infection, we generated bone marrow chimeras where B6 wild-type bone marrow was grafted into either wild-type or IFNAR1/IL28Rα deficient hosts. These two groups have both fully functional immune cells but differ in the ability of radioresistant cells including lung epithelia to respond to type I and III IFNs. We confirmed successful immune cell reconstitution by staining blood cells for IFNαβR (Fig. S6) and by testing Sca-1 upregulation on blood cells in response to IFNβ (not shown). Both experiments confirmed that >85% of immune cells in these chimeras have wt phenotype. When these chimeras were infected with the PR8 strain, high susceptibility and mortality was found only in the group lacking IFN receptors on stromal cells and this correlated with higher viral titers (Fig. 6A, B). These results suggest that the ability to respond to IFNs in infected airway epithelia is crucial for successful elimination of the virus from infected animals.

### A residual production of IFNs is responsible for ISG induction in MAVS^−/−^ epithelia

Infected MAVS^−/−^ and IRF3^−/−^IRF7^−/−^ epithelia show ISG induction in the absence of any detectable IFN upregulation, while IFNAR1^−/−^IL-28Rα^−/−^ cells, which produce but cannot respond to IFNs, did not express ISGs in response to infection. Although type I IFN genes are tightly regulated in response to viral infection, many tissues constitutively secrete low amounts of type I IFN even in the absence of infection (reviewed in [28]). It has been proposed that these constitutive levels of IFNs are required to maintain basal expression of IFN-inducible signalling intermediates (STAT1/2, IRF7/9/5) and to modulate the relative expression of STAT proteins, therefore “priming” cells for future responses.

**Figure 7:**
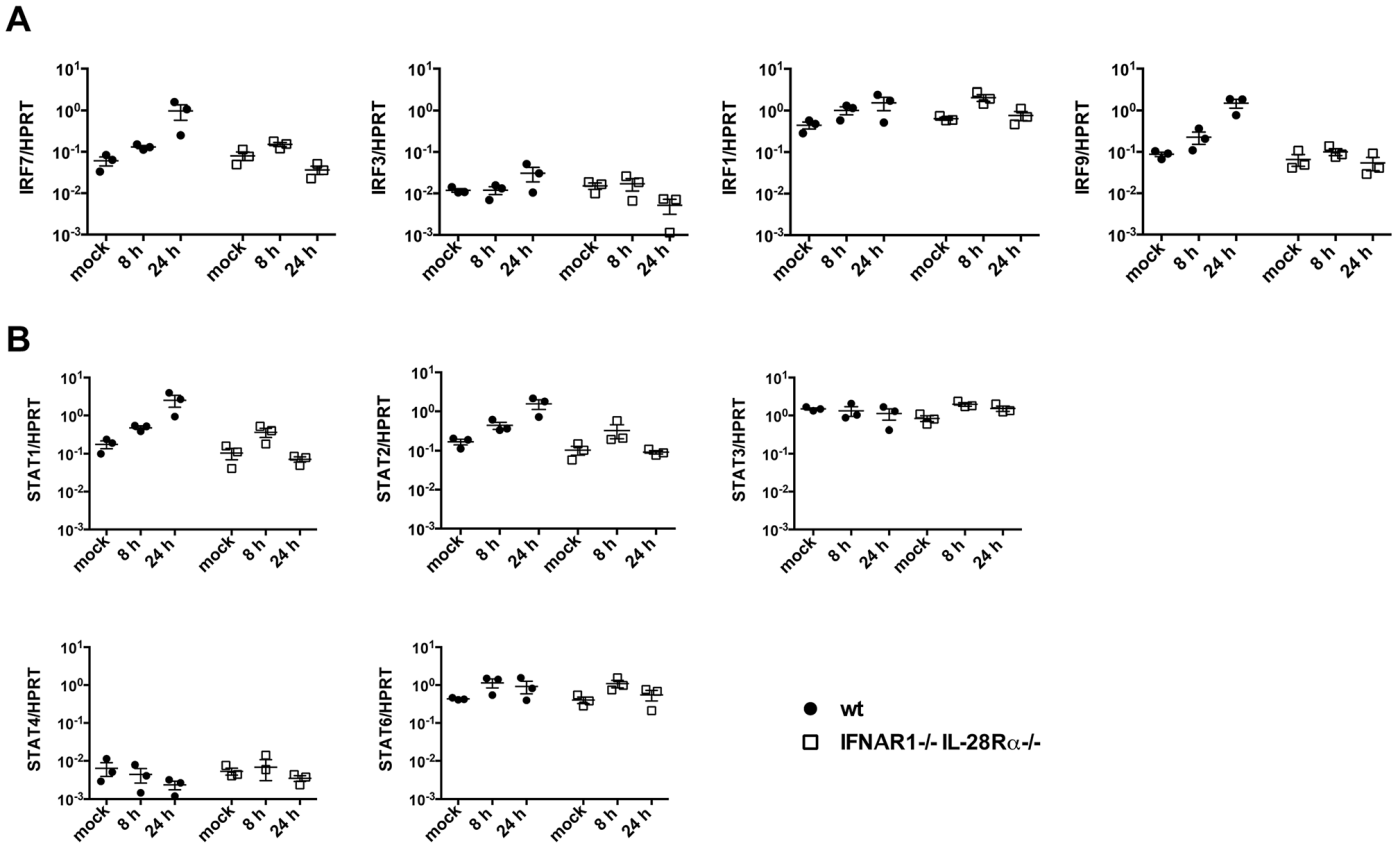
Basal expression of signalling intermediates is similar in wild-type and IFNAR1^−/−^IL-28Rα^−/−^ double knock-out MTEC. (A) Relative expression of IRF3, IRF1, IRF7 and IRF9 was determined in both mock infected and PR8 infected MTEC by qRT-PCR. The transcripts quantities are expressed as ratio to HPRT levels +/− SEM. (B) The same RNA samples were analyzed for STAT1, STAT2, STAT3, STAT4 and STAT6 expression.

For these reasons, we sought to determine the basal level of different IFN-signalling intermediates at steady state and upon infection in IFNAR1^−/−^IL-28Rα^−/−^ cells. Wild-type and double knock-out cells were infected with influenza A and the level of different STAT and IRF molecules analyzed by qPCR. Some of these molecules (IRF7, IRF9, STAT1, STAT2) are known ISGs and were upregulated in wild-type but not in IFNAR1^−/−^IL-28Rα^−/−^ epithelia at 24 hours post infection. However, the levels of all these transcripts measured relatively to HPRT at steady state were comparable in the two genotypes (Fig. 7A, 7B) and in MAVS^−/−^ and IRF3^−/−^IRF7^−/−^ cells (not shown).

**Figure 8:**
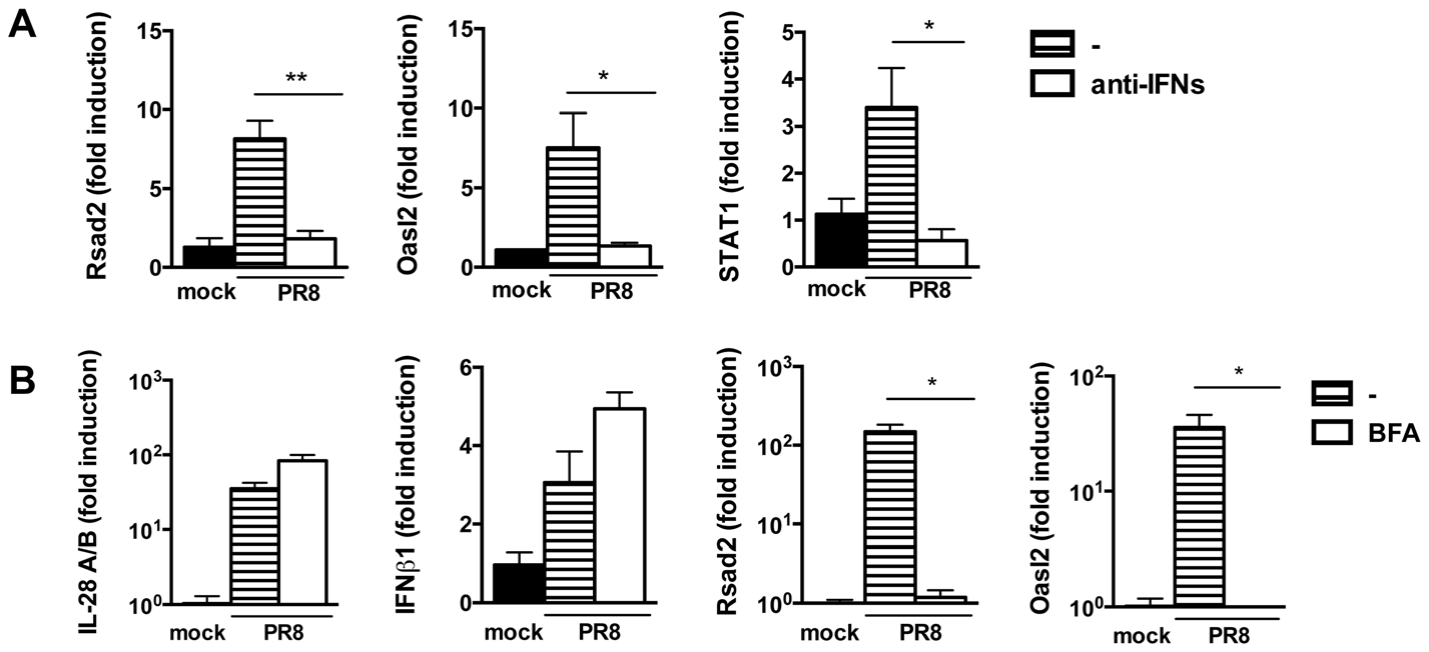
Residual IFN production is responsible for ISG induction in MAVS−/− epithelia. (A) qRT-PCR analysis of Rsad2, Oasl2 and STAT1 in MAVS−/− epithelia infected with PR8 in the presence or absence of blocking anti-IFNAR and neutralizing anti-IL28A/B antibodies. (B) Wild-type epithelia were PR8-infected in the presence or absence of brefeldin A (2.5 µg/ml). Expression of the indicated genes was determined by qRT-PCR at 24 hpi. All transcripts were normalized to HPRT levels and then expressed as fold induction relative to the mean of mock infected controls, +/− SEM. Asterisks indicate differences that are statistically significant (unpaired t test; *, P<0.05; **, P<0.01).

To test directly whether residual IFN production is responsible for ISG induction in MAVS and IRF3/7 deficient epithelia, we compared ISG induction in infected MAVS deficient epithelia in the presence or absence of an antibody cocktail blocking both IFNαβ and IFNλ signalling. As shown in Figure 8A, antibody-treated epithelium had further reduced ISG expression compared to the untreated one. To confirm independently the requirement of autocrine signalling by soluble factors for ISG induction, we infected wild-type epithelia in the presence or absence of brefeldin A (BFA). BFA treatment left IFN gene induction unaffected but abolished ISG induction, indicating that soluble factors which include IFNs are required for ISG induction (Fig. 8B).

Collectively, these results indicate that the different responses to infection in MAVS^−/−^ and IRF3^−/−^IRF7^−/−^ compared to IFNAR1^−/−^IL-28Rα^−/−^ cells can not be ascribed to a lack of priming in the latter, but are most likely due to a residual production of IFN in MAVS^−/−^ and IRF3^−/−^IRF7^−/−^ cells, that, in the context of infection, is sufficient to ensure ISG induction.

## Discussion

Here, we delineate the influenza-triggered pathways leading to the induction of an antiviral state in primary airway epithelia, the first and most important target tissue of the virus in an infected organism. We show that TLR7 or other TLRs relying on the adaptor molecules MyD88 and TRIF are not involved in the induction of interferons, while the RIG-I/MAVS pathway is crucial for this process. We also show that between the two transcription factors IRF3 and IRF7 implied in IFN induction, IRF3 is of less importance than IRF7, but complete abolition of influenza-triggered IFN expression is seen only in the absence of both molecules. Most importantly, we show that, upon influenza infection, IFN type I and III independently mediate parallel amplification loops leading to the induction of a completely overlapping set of ISGs, and that this induction is abolished only when none of the two amplification loops are active.

The general paradigm for type I IFN induction involves recruitment of transcription factors that are activated by phosphorylation in response to signalling cascades stimulated during viral infection. The IFNβ promoter contains four positive regulatory domains (PRDI-IV), which are occupied by different transcription factors. PRDI and III are binding sites for IRF3 (early during infection, due to its constitutive expression) and IRF7 (with delayed kinetics, due to its inducible expression through an IFN-dependent positive feedback loop), while PRDIV and PRDII bind the ATF-2/c-Jun AP-1 and the p50/RelA NF-κB complexes respectively (reviewed in [8]).

This initial model of a positive feedback loop, in which IRF3 is primarily responsible for the early induction of IFNβ while IRF7 is required later in the response [29], was subsequently modified when a study performed on IRF7^−/−^ MEFs revealed that both the early and the late production of type I IFN induced by VSV or EMCV is abolished in the absence of IRF7 [30]. Our results are in line with these observations and identify IRF7 as the major regulator of both type I and type III IFN responses in epithelial cells. Indeed, our data in fig. 6A suggests that the expression level of IRF7 was higher than that of IRF3 at steady state, which would support the notion that IRF7 could act directly downstream of viral recognition to induce IFNs at the earliest stage of infection. Moreover, our data indicates that, even later during an infection, the expression of IFNβ1 and IL-28A/B can be sustained independently of the IFN-driven positive amplification loop (Fig. 5B).

IFNλ is preferentially induced by influenza A virus, both in vivo and in vitro [31]. We extend these findings to primary airway epithelia and demonstrate that epithelial-derived IFN type I or type III are sufficient to fuel their respective amplification loop and that no extrinsic IFNs from other cells, for instance immune cells, is required to induce an epithelial IFN signature.

The importance of type III IFNs in epithelial responses has been well documented. Several studies have shown that IFNλ protects the epithelium of lung, intestine and vagina from viral infections and that IFNAR1^−/−^IL-28Rα^−/−^ double deficient mice are more susceptible to viral infection than each single knock-out strain [17], [18], [32]. Using chimeric mice with a wild type immune system and either wild type or IFNAR1^−/−^IL-28Rα^−/−^ double deficient stroma, we show here that IFN unresponsiveness in the stromal cell compartment is sufficient to render mice more susceptible to influenza infection.

Previous studies have shown that the promoters of many ISGs have a simple structure and can be easily turned on directly by IRF proteins independently of interferon. In these studies, alternative pathways of direct ISG induction were suggested that rely on either IRF3 [23], IRF7 [25], IRF1 [26] or peroxisomal MAVS/IRF1/IRF3 [33]. More recently, the cytosolic exonuclease Trex1 has also been identified as a negative regulator of a novel pathway involving STING, TBK1, IRF3 and IRF7 that can lead to interferon-independent activation of ISGs [34].

The finding that a subset of ISGs could still be induced in both MAVS- and IRF3/IRF7-deficient epithelia suggested to us that IFN-independent ISG induction may take place here. However, the nearly complete absence of ISG induction in IFNAR1^−/−^IL-28Rα^−/−^ epithelia led us to conclude that, at least in our experimental model, no other mechanism can efficiently replace the IFN loop for the induction of ISGs. Constitutive low-level signalling of IFNβ (IFN “priming”) has been suggested to help preserve IFN responsiveness but also to allow IFN-independent ISG induction [28], by maintaining the expression of STATs and other signalling intermediates. It could be argued that, unlike IFNAR1^−/−^IL-28Rα^−/−^ epithelia, MAVS^−/−^ and IRF3^−/−^IRF7^−/−^ cells still possess this sub-threshold signalling which helps maintain STATs and IRFs at sufficient levels to allow for direct ISG induction upon viral trigger, even in the absence of IFNs. We did however not detect steady-state differences in the expression of a range of IRF and STAT molecules between genotypes and therefore have no evidence that differences in IFN priming contribute to the phenomenon described here.

Previous studies that assessed IFN independent ISG induction have mostly relied on IFNAR deficient cells to confirm IFN independence. Here we show that care must be taken to evaluate IFN independence. As IFN type III can stand in for IFN type I in inducing an IFN signature, the analysis of each single receptor knock-out epithelium would have wrongly suggested complete independence of ISG induction from the IFN system. Moreover, while the IFN signature was completely abolished in influenza infected IFNAR1^−/−^IL-28Rα^−/−^ cells, the addition of neutralizing antibodies against secreted type I and type III IFNs, used in combination on wild-type epithelia or for the complementary IFN on IFN receptor single knock-out epithelia, had little effect on ISG induction (not shown), indicating that even minute concentrations of IFN were still able to induce ISG expression in responsive cells.

In vivo studies with single-knock-out mice clearly showed that ISG induction by type III IFN translates into less powerful protection against influenza A virus than ISG induction by type I IFN [32]. At present it is unclear whether slight differences in the kinetics of virus-triggered induction of type I and type III IFN may account for this observation. An alternative explanation is that lung macrophages which are productively infected by most influenza A virus strains and which do not respond well to type III IFN may quickly amplify the incoming virus in the respiratory tract of IFNAR1-deficient mice and thus overwhelm the type III-mediated protection of epithelial cells in such mice.

Through the induction of ISGs, the IFN system has potent effects not only to directly combat virus, but also on cell physiology and survival and on the immune response. Therefore, it is considered a very tightly controlled system to avoid excessive inflammation, cell death and tissue damage. On this background, we were surprised to find that in MAVS and IRF3/7 deficient epithelia, IFNs at levels below ELISA or qPCR detection threshold still lead to only slightly reduced ISG upregulation. ISG induction disappeared in MAVS deficient epithelia when IFN type I and III signalling was blocked by an antibody cocktail, indicating that a residual IFN production drives ISG induction in these cells.

In apparent contrast to these results, when exogenous IFN was titrated onto epithelial cultures, the minimum amount of IFN required to induce ISG expression was clearly detectable by the ELISA assay (not shown). Possible reasons for this discrepancy could be differences in the bioactivity of endogenous versus recombinant IFNs and differential ability by the ELISAs to detect endogenous or recombinant IFNs. Moreover, biological explanations include local concentrations of endogenous IFNs that may be much higher than those measured in the total supernatant, and the possibility that autocrine IFNs bind to their receptor already in intracellular vesicles and are therefore not measured by ELISA.

Overall these data suggest that presence or absence, rather than absolute amounts of IFN, determine the response. The biological sense of such a binary switch could be to respond robustly even to small perturbations of the steady state, which may gain the host precious time when infected by fast-replicating viruses. In the uninfected state, absolute IFN shut-down would be required to avoid chronic “Flu-like symptoms”, a scenario that is in contradiction to the proposed priming effect of sub-threshold IFN levels.

One hypothesis for how the vast differences in IFN levels are translated into a largely unaltered IFN signature in MAVS^−/−^ and IRF3^−/−^IRF7^−/−^ epithelia is that Influenza A infection may render cells much more IFN-sensitive by unknown pathways. To test this hypothesis, we titrated exogenous IFN on uninfected or infected wt or MAVS-deficient epithelia and measured ISG induction. No increased IFN responsiveness was found in infected versus uninfected epithelia, suggesting that there is no synergy between these two signals (not shown). An alternative hypothesis is that IFN protein that is prestored [35] and therefore not measurable by qPCR (not transcriptionally controlled) or by ELISA (too low sensitivity) could be released locally and mediates the observed ISG induction.

In conclusion, we show here that airway epithelia rely on two parallel, redundant amplification loops to induce an IFN signature in response to influenza A infection. Only a small fraction of genes, mostly non ISGs, are induced by the virus in the absence of both IFN systems, and no ISG appears to rely specifically on one IFN system only. This complete redundancy may guarantee induction of antiviral responses even if one or the other IFN system is blocked, for instance by specific virally encoded antagonists. In contrast to the two redundant IFN loops in epithelia, the majority of immune cells respond only to IFN type I, thus potentially allowing for differential control of the epithelial antiviral state and the induction of immune responses: while high levels of IFN type I would activate both epithelia and immune cells, high levels of type III would specifically activate epithelial responses but leave immune responses unaffected, which may help limit immune-mediated pathology in the lung and at other mucosal surfaces.

## Materials and Methods

### Ethics statement

All animal breeding was approved by the local ethical committee of the NIMR and is part of a project approved by the UK Home Office (licence number 80/2236). Breeding was conducted according to local guidelines and UK Home Office regulations under the Animals Scientific Procedures Act 1986 (ASPA).

### Viruses

Influenza A virus strain A/PR/8/34 (H1N1) was grown in day 10 embryonated chicken eggs, and titrated on MDCK by 50% tissue culture infective dose (TCID_50_), according to the Spearman-Karber method.

### Mice

All cells used in this study were derived from mice on the C57BL/6 background. MAVS^−/−^ mice [36] and tracheae from TLR7^−/−^ mice [37] were kindly provided by Dr. C. Reis e Sousa; MyD88^−/−^ [38], IFNAR1^−/−^ [39] and TRIF^−/−^ mice [40] were kindly provided by Dr. A. O'Garra. These and C57BL/6 wt mice were bred in-house under SPF conditions. Tracheae from Irf3^−/−^ [29]; Irf7^−/−^ [29]; Irf3^−/−^Irf7^−/−^ cells were also used. IL-28Rα^−/−^ [12], IFNAR1^−/−^ [39] and IL-28Rα^−/−^IFNAR1^−/−^ cells were obtained from mice on a congenic B6.A2G-Mx1 background carrying an intact Mx1 gene [41].

To generate chimeric mice, naïve B6.A2G-Mx1 and B6.A2G-Mx1 IL-28Rα^−/−^IFNAR1^−/−^ recipient mice were lethally irradiated with 1000 rad and reconstituted with donor B6.A2G-Mx1 BM cells (7×10^6^) by intravenous injection. Chimeric mice were maintained for 7 weeks and chimerism assessed by IFNAR1 (MAR1-5A3 antibody) expression on Gr1+, CD19+, CD4+ and CD8+ cells in the blood (Fig. S6). Chimeric mice were then infected intranasally with 10^5^ TCID_50_ of Influenza A/PR/8/34 in 30 µl PBS after anesthesia.

### Primary mouse tracheal epithelial cell culture (MTEC)

Isolation and culture of primary MTEC were performed as previously described [42]. Briefly, cells isolated by enzymatic treatment were seeded onto 0.4 µm pore size clear polyester membrane (Corning) coated with a collagen solution. At confluence, media was removed from the upper chamber to establish an air- liquid interface (ALI). Fully differentiated, 10–14 days-old post ALI cultures were routinely used for experiments.

In some experiments, cultures were infected in the presence of brefeldin A (2.5 µg/ml) or neutralizing anti-IL28A/B, anti-IL28B (R&D Systems) and blocking anti-IFNAR1 (MAR1-5A3) antibodies, at a final concentration of 10 µg/ml each.

### Immunofluorescence microscopy

Differentiated, ALI day 14 cultures were fixed in 4% paraformaldehyde and permeabilized with 0.1% Triton X-100. Cells were then incubated with the indicated primary antibodies for 1 hour at room temperature, washed, incubated with fluorochrome-conjugated secondary antibody, washed and finally mounted. Image acquisition and processing information: (i) microscope: Olympus IX70; (II) magnification: 20×; (III) imaging medium: Vectashield with DAPI (Vector labs); (IV) fluorochromes: Alexa Fluor 488, Alexa Fluor 568. (V) acquisition software: Softworx. Images were processed with Image J.

### Virus infection

The apical surface of MTEC cultures was washed extensively to remove accumulated mucins before inoculation with IAV (moi = 0.3). After incubation at 37°C for 1 h, the virus inoculum was removed and the cultures were incubated in complete growth medium for 24 hours. Aliquots of the supernatants were collected at different time points and titrated by ELISA as described below. Cells were then lysed to extract RNA or for detection of viral protein by Western blotting.

### RNA extraction and real time-PCR

RNA was isolated from MTEC cultures by directly lysing the cells in the transwells, using the Qiagen RNeasy mini kit, according to the manufacturer's instructions. One microgram total RNA was reverse transcribed using the ThermoScript RT-PCR System kit (Invitrogen). The cDNA served as template for the amplification of genes of interest and the housekeeping gene (*Hprt1*) by real-time PCR, using TaqMan Gene Expression Assays (Applied Biosystems), universal PCR Master Mix (Applied Biosystems) and the ABI-PRISM 7900 sequence detection system (Applied Biosystems). The fold increase in mRNA expression was determined using the ΔΔC_t_ method relatively to the values in mock treated samples, after normalization to *Hprt1* gene expression.

### Microarray data analysis

Total RNA harvested from MTEC cultures was hybridized using Affymetrix Mouse Genome 430 2.0 microarrays. The raw intensities values for each entity were preprocessed by RMA normalization against the median intensity in mock infected samples. Using GeneSpring 11.5, all transcripts were filtered based on signal values, to select the ones whose level of expression was in the 100–20^th^ percentile, in at least 50% of samples. Student's t test (infected versus mock infected) or 2-way ANOVA (parameters: treatment and genotype) were performed to identify gene significantly differentially expressed relative to controls (≥4-fold change; p<0.01, Benjamini-Hochberg multiple test correction).

Ingenuity Pathway Analysis (IPA) was used to select, annotate and visualize gene by function and pathway. ISGs were identified with the Interferome database (www.interferome.org/).

Microarray data can be accessed at GEO under accession number GSE43710 for the superseries.

### Quantification of protein by ELISA

Cell culture supernatants were harvested from the apical compartments of mock or IAV infected samples. IL-28A/B was measured using the IL-28A/B ELISA Duo kit (R&D Systems), IFNβ with the Verikine IFNβ ELISA kit (PBL Interferon Source).

## Supporting Information

Figure S1 Flow cytometric measurement of influenza infection of epithelial cells. Fully differentiated MTEC cultures were infected with A/PR/8/34 (H1N1) (moi = 0.3). At 24 hours post infection, cell were washed, trypsinized to get single cell suspension and then fixed in 4% paraformaldehyde. After permeabilization, cells were stained with anti-NP/M-FITC antibodies (Imagen Oxoid) and then analysed by flow cytometry.
(TIF)

Figure S2 Heat inactivation blocks virus replication but still permits virus binding to cells. (A) Fully differentiated MTEC cultures were infected with either PR8 or PR8 (HI) (heat inactivated at 65°C) (moi = 3). At the indicated time point, cells were washed and lysed in 1% Triton X-100. Cell extracts were then run on a polyacrylamide gel, transferred to nitrocellulose membrane and probed with specific antibodies plus horseradish peroxidase-conjugate secondary antibody (Biorad). The early appearance of influenza Matrix protein (M) testifies binding of the virions to the target cells; the later positiveness for influenza NS is indicative of viral replication. (B) MTEC cultures were infected with PR8 at a moi = 0.3. 36 hours post infection supernatants from the apical side were collected and titrated on MDCK. Virus titres were expressed as tissue culture infectious dose 50 (TCID_50_).
(TIF)

Figure S3 Transcriptional response to influenza infection in the absence of MAVS. Total RNA from mock and PR8 infected wild-type and MAVS^−/−^ cells was analysed using Affymetrix Mouse Genome 430 2.0 microarrays, at 24 hpi. The raw intensities values for each entity were processed by RMA normalization against the median intensity in mock infected samples. All transcripts were filtered based on signal values, to select the ones whose level of expression was in the 100–20^th^ percentile, in at least 50% of samples. Supervised analysis was performed using statistical filtering (≥4-fold change relative to mock infected wild-type in at least one treatment group; 2-way ANOVA, p<0.01, Benjamini-Hochberg multiple test correction). (A) K-means clustering of the differentially expressed genes. (B) Heat map of the upregulated genes shown in (A). The range of fold changes is expressed in a log2 scale.
(TIF)

Figure S4 Identification of genes induced independently of IFN type I or type III. Total RNA from mock and PR8 infected cells was analysed using Affymetrix Mouse Genome 430 2.0 microarrays at 24 hpi. Supervised analysis was performed as in figure 5A, using statistical filtering (≥4-fold change relative to mock infected wt; 2-way ANOVA, p<0.01, Benjamini-Hochberg multiple test correction). The upregulated genes shown in figure 5A were further partitioned by K-means clustering to generate two clusters of which the one shown in (A) includes the genes that were upregulated upon infection in all genotypes. (B) shows the same cluster as a heat map. The cluster in (C) includes the 110 genes that were upregulated only in wild-type and single knock-out epithelia. 58 genes of the latter list were recognized as ISG by the INTERFEROME database. The range of fold changes is expressed in a log2 scale.
(TIF)

Figure S5 Assessment of genes differentially expressed in the absence of either IFN type I or type III. Total RNA from mock and PR8 infected cells was analysed using Affymetrix Mouse Genome 430 2.0 microarrays at 24 hpi. Supervised analysis was performed as in figure 5A, using statistical filtering (≥4-fold change relative to mock infected wt; 2-way ANOVA, p<0.01, Benjamini-Hochberg multiple test correction). The upregulated genes described in figure 5A were further filtered (≥4-fold change in expression for corresponding time points for the following pairs of condition: IFNAR1^−/−^ versus wild-type; IL-28Rα^−/−^ versus wild-type; IL-28Rα^−/−^ versus IFNAR1^−/−^) to generate the heat map shown here.
(TIF)

Figure S6 Confirmation of successful bone marrow grafting in chimera experiments. Seven weeks after BM graft, mice were bled from the tail vein. Blood was collected in heparin and stained with the following antibodies: CD19 (6D5), Gr-1 (1A8), CD4 (RM4-5), CD8 (53-6.7) and IFNAR1 (MAR1-5A3). Samples were read by FACSCanto (BD Bioscience) and analyzed by FlowJo 9.6.2. Cells from wild-type C57BL/6 (solid grey), IFNAR1−/− (black line) and chimeric mice (coloured lines) are shown.
(TIF)
