# Supplementary figures and images for: Type I and Type III Interferons Drive Redundant Amplification Loops to Induce a Transcriptional Signature in Influenza-Infected Airway Epithelia
Source: PLoS Pathog. 2013 Nov 21;9(11):e1003773. doi: 10.1371/journal.ppat.1003773 (PMC3836735; doi:10.1371/journal.ppat.1003773)

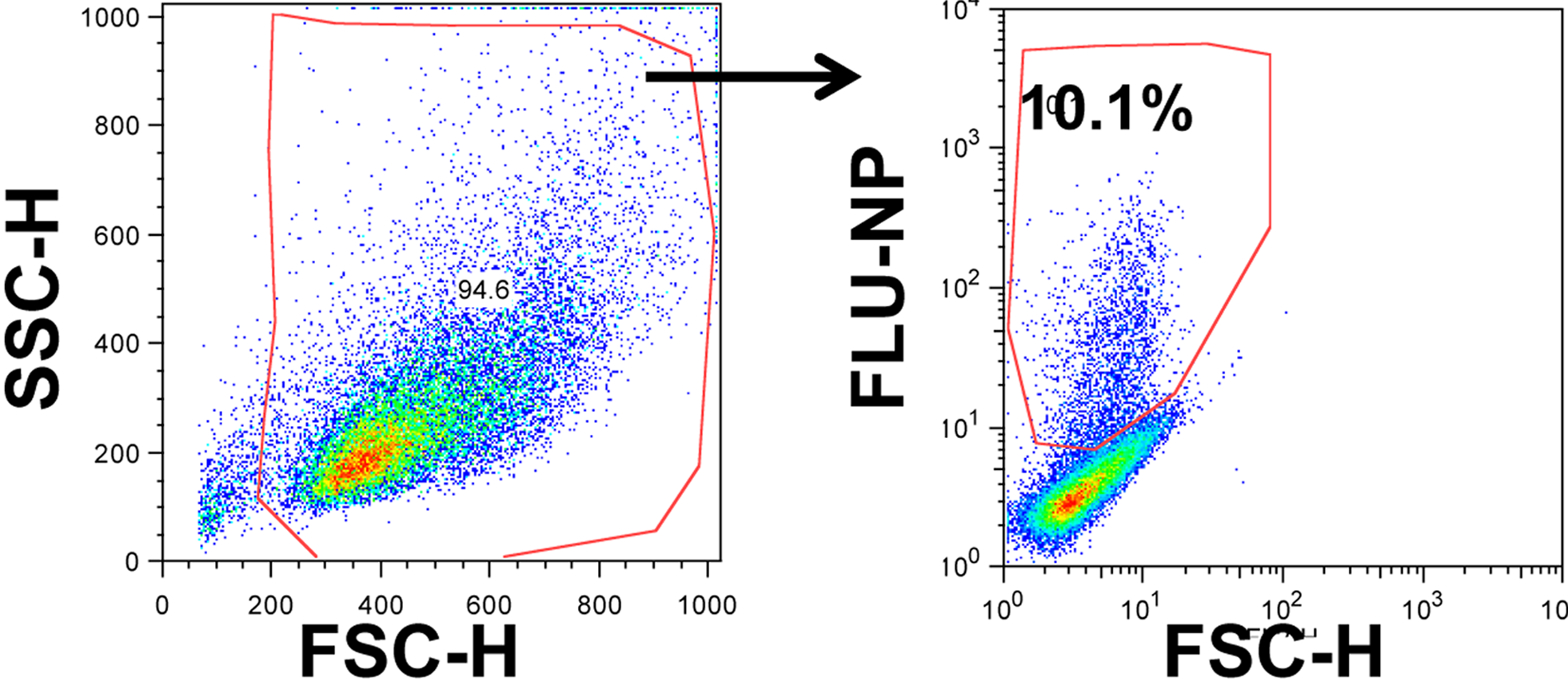

Supplement: Figure S1 — Flow cytometric measurement of influenza infection of epithelial cells. Fully differentiated MTEC cultures were infected with A/PR/8/34 (H1N1) (moi = 0.3). At 24 hours post infection, cell were washed, trypsinized to get single cell suspension and then fixed in 4% paraformaldehyde. After permeabilization, cells were stained with anti-NP/M-FITC antibodies (Imagen Oxoid) and then analysed by flow cytometry. (TIF) [file ppat.1003773.s001.tif]

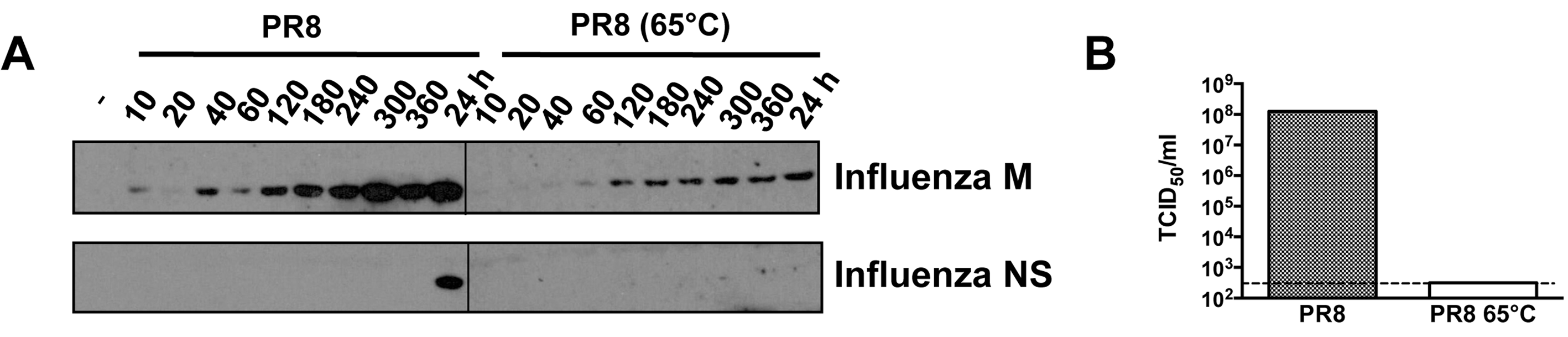

Supplement: Figure S2 — Heat inactivation blocks virus replication but still permits virus binding to cells. (A) Fully differentiated MTEC cultures were infected with either PR8 or PR8 (HI) (heat inactivated at 65°C) (moi = 3). At the indicated time point, cells were washed and lysed in 1% Triton X-100. Cell extracts were then run on a polyacrylamide gel, transferred to nitrocellulose membrane and probed with specific antibodies plus horseradish peroxidase-conjugate secondary antibody (Biorad). The early appearance of influenza Matrix protein (M) testifies binding of the virions to the target cells; the later positiveness for influenza NS is indicative of viral replication. (B) MTEC cultures were infected with PR8 at a moi = 0.3. 36 hours post infection supernatants from the apical side were collected and titrated on MDCK. Virus titres were expressed as tissue culture infectious dose 50 (TCID50). (TIF) [file ppat.1003773.s002.tif]

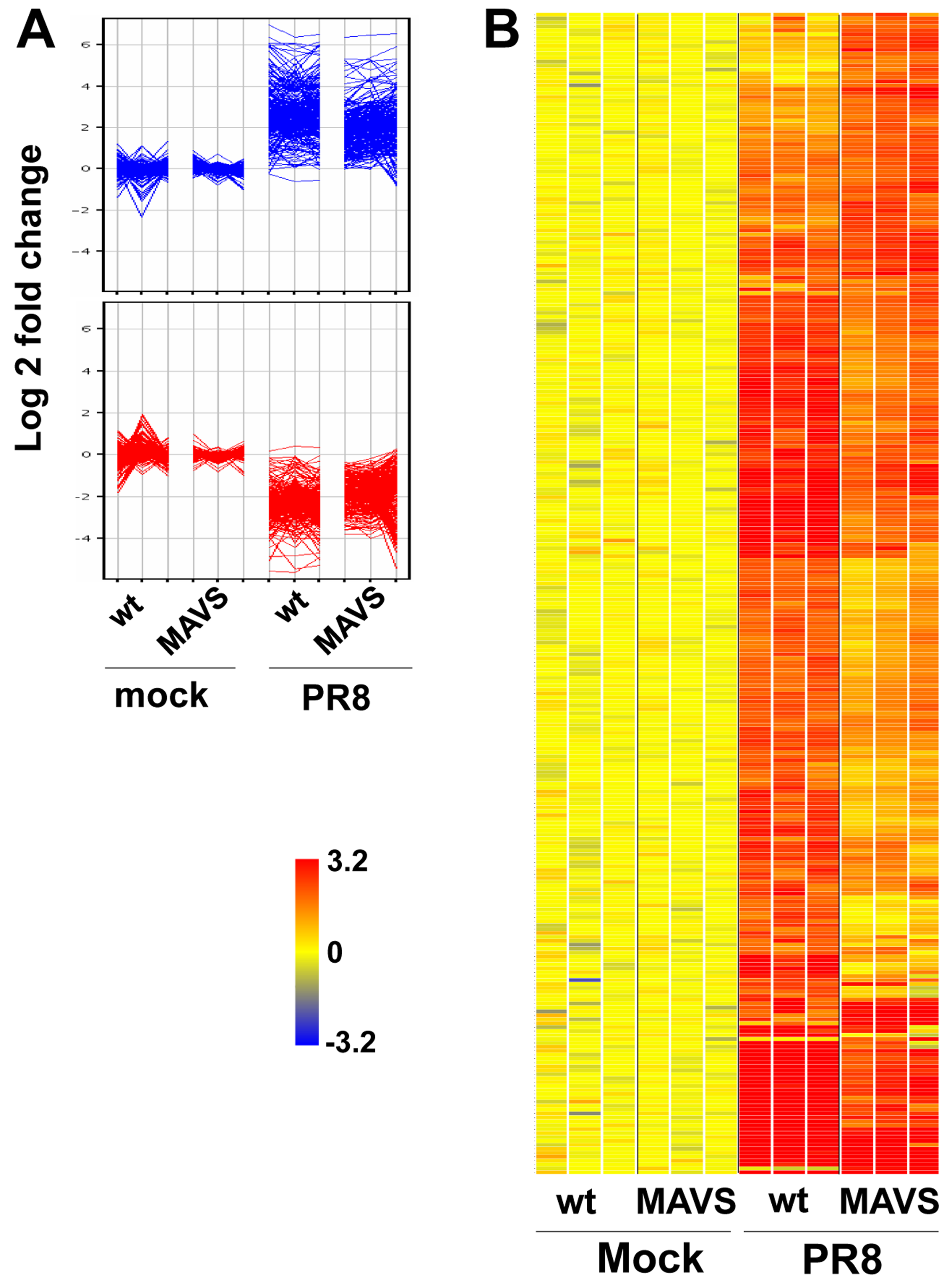

Supplement: Figure S3 — Transcriptional response to influenza infection in the absence of MAVS. Total RNA from mock and PR8 infected wild-type and MAVS−/− cells was analysed using Affymetrix Mouse Genome 430 2.0 microarrays, at 24 hpi. The raw intensities values for each entity were processed by RMA normalization against the median intensity in mock infected samples. All transcripts were filtered based on signal values, to select the ones whose level of expression was in the 100–20th percentile, in at least 50% of samples. Supervised analysis was performed using statistical filtering (≥4-fold change relative to mock infected wild-type in at least one treatment group; 2-way ANOVA, p<0.01, Benjamini-Hochberg multiple test correction). (A) K-means clustering of the differentially expressed genes. (B) Heat map of the upregulated genes shown in (A). The range of fold changes is expressed in a log2 scale. (TIF) [file ppat.1003773.s003.tif]

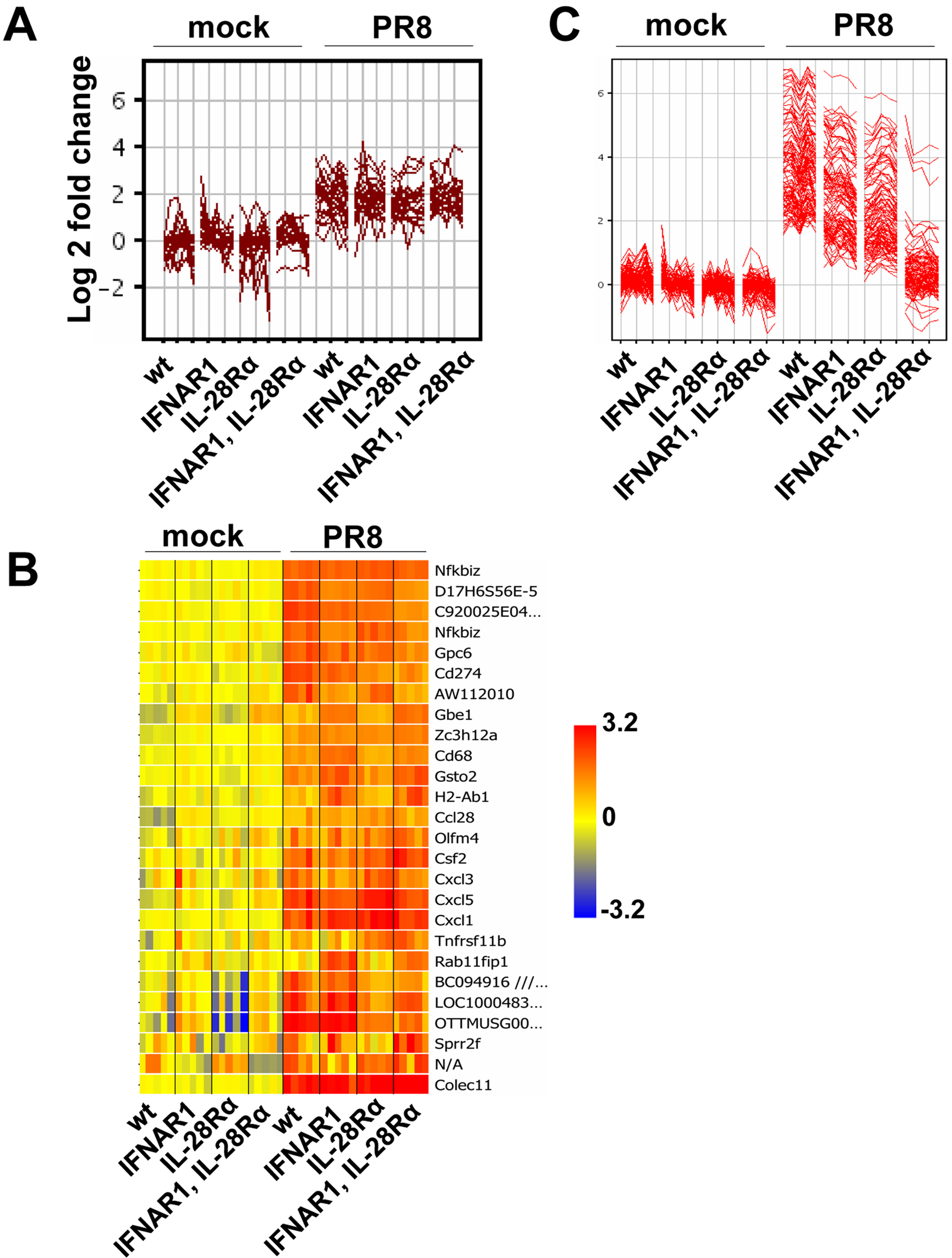

Supplement: Figure S4 — Identification of genes induced independently of IFN type I or type III. Total RNA from mock and PR8 infected cells was analysed using Affymetrix Mouse Genome 430 2.0 microarrays at 24 hpi. Supervised analysis was performed as in figure 5A, using statistical filtering (≥4-fold change relative to mock infected wt; 2-way ANOVA, p<0.01, Benjamini-Hochberg multiple test correction). The upregulated genes shown in figure 5A were further partitioned by K-means clustering to generate two clusters of which the one shown in (A) includes the genes that were upregulated upon infection in all genotypes. (B) shows the same cluster as a heat map. The cluster in (C) includes the 110 genes that were upregulated only in wild-type and single knock-out epithelia. 58 genes of the latter list were recognized as ISG by the INTERFEROME database. The range of fold changes is expressed in a log2 scale. (TIF) [file ppat.1003773.s004.tif]

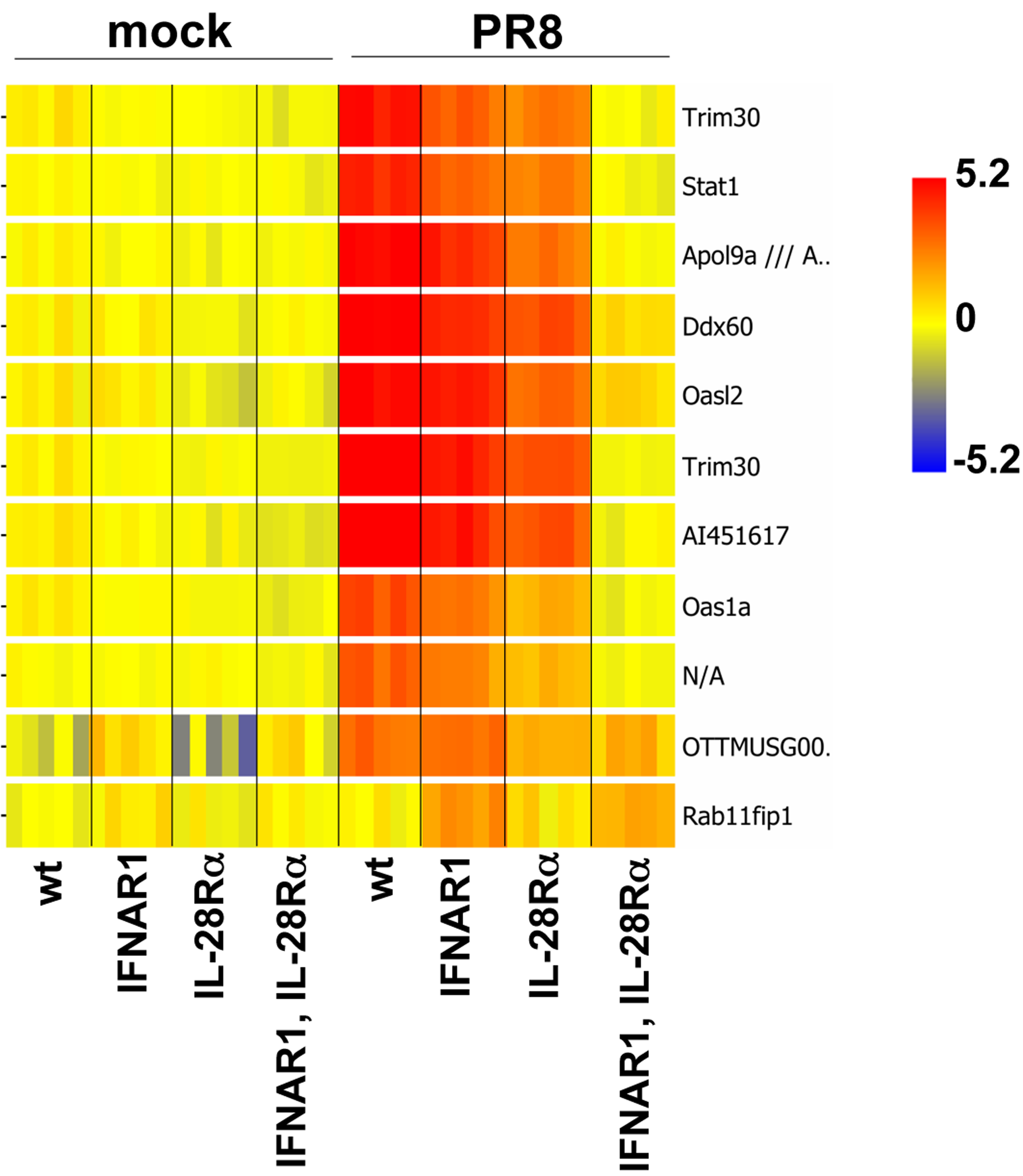

Supplement: Figure S5 — Assessment of genes differentially expressed in the absence of either IFN type I or type III. Total RNA from mock and PR8 infected cells was analysed using Affymetrix Mouse Genome 430 2.0 microarrays at 24 hpi. Supervised analysis was performed as in figure 5A, using statistical filtering (≥4-fold change relative to mock infected wt; 2-way ANOVA, p<0.01, Benjamini-Hochberg multiple test correction). The upregulated genes described in figure 5A were further filtered (≥4-fold change in expression for corresponding time points for the following pairs of condition: IFNAR1−/− versus wild-type; IL-28Rα−/− versus wild-type; IL-28Rα−/− versus IFNAR1−/−) to generate the heat map shown here. (TIF) [file ppat.1003773.s005.tif]

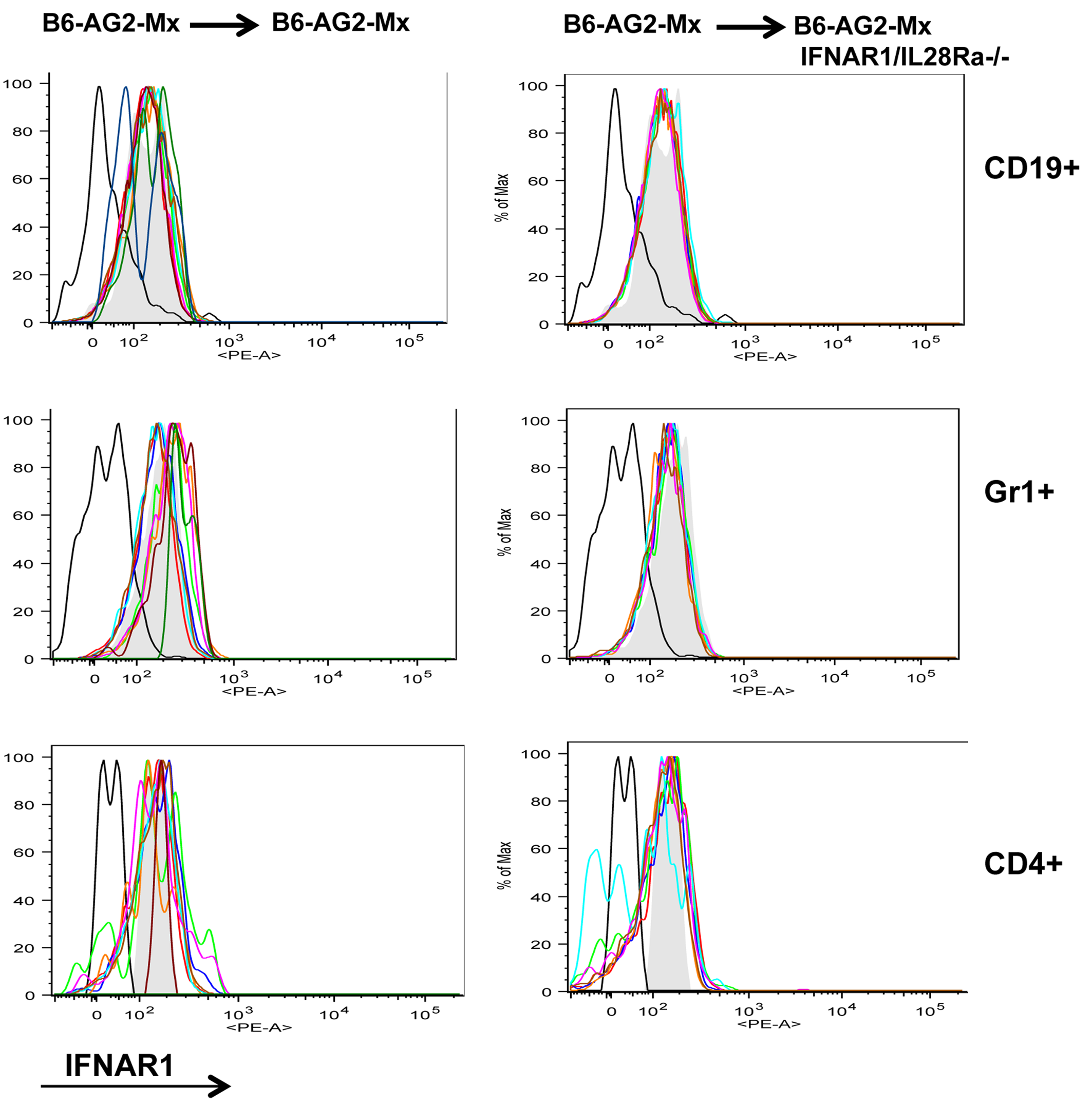

Supplement: Figure S6 — Confirmation of successful bone marrow grafting in chimera experiments. Seven weeks after BM graft, mice were bled from the tail vein. Blood was collected in heparin and stained with the following antibodies: CD19 (6D5), Gr-1 (1A8), CD4 (RM4-5), CD8 (53-6.7) and IFNAR1 (MAR1-5A3). Samples were read by FACSCanto (BD Bioscience) and analyzed by FlowJo 9.6.2. Cells from wild-type C57BL/6 (solid grey), IFNAR1−/− (black line) and chimeric mice (coloured lines) are shown. (TIF) [file ppat.1003773.s006.tif]
